# Single‐Cell Transcriptome Reveals Aquaporin‐Mediated Carbon Nanosol‐Induced Growth Promotion of Plants

**DOI:** 10.1002/advs.202504459

**Published:** 2025-04-30

**Authors:** Lingtong Cheng, Zechao Qu, Qiansi Chen, Lin Wang, Huan Su, Jiemeng Tao, Peng Lu, Taibo Liang, Jianfeng Zhang, Peijian Cao, Jingjing Jin

**Affiliations:** ^1^ Beijing Life Science Academy Beijing 102200 China; ^2^ China Tobacco Gene Research Center Zhengzhou Tobacco Research Institute of CNTC Zhengzhou 450001 China; ^3^ Key Laboratory of Ecological Environment and Tobacco Quality Zhengzhou Tobacco Research Institute of CNTC Zhengzhou 450001 China

**Keywords:** aquaporins, carbon nanosol, developmental trajectories, plant growth, scRNA‐seq

## Abstract

Carbon nanosol (CNS) is a novel carbon nanomaterial with the potential for enhancing plant growth, yet the underlying mechanism remains unclear. Application of 10 mg L^−1^ CNS significantly promotes plant growth, increasing fresh mass by 59.51%. Track of fluorescent labeling CNS reveals that it is rapidly absorbed by roots and entered the vascular bundle and cortex within 2 h. A single‐cell transcriptomic atlas of tobacco roots response to CNS treatment is generated, which comprises 7,897 cells representing 13 distinct cell types. CNS is found to affect gene expression in a cell type‐specific manner, suggesting the heterogeneity of plant response to CNS. Further pseudo‐time trajectory analysis reveals that most cell types undergo cell fate transitions toward a more mature state under CNS treatment. In addition, aquaporin proteins NIPs and TIPs are found to be activated and significantly upregulated in epidermal, cortical, and endodermal cells. Further genetic and physiological findings reveal that growth enhance effect of CNS for *tip1;1* and *tip2;1* mutants is significantly weakened compared to the wild type, indicating that aquaporins play an important role in CNS‐mediated plant growth promotion. Overall, these results provide new insights into the mechanism by which CNS promotes plant growth at the single‐cell level.

## Graphical abstract

Carbon nanosol (CNS) exhibits rapid root penetration capacity, enhancing plant growth through boosting stomatal density and antioxidative enzyme activity. Single‐cell transcriptomic profiling uncovers cell type‐specific gene expression patterns and developmental trajectories during CNS‐mediated growth promotion. Notably, growth enhance effect of CNS for tip1;1 and tip2;1 mutants was significantly weakened compared to the wild type, indicating that aquaporins play an important role in CNS‐mediated plant growth promotion.

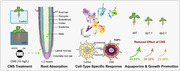

## 1 Introduction

Nanomaterials (NMs) have been widely applied in the fields of agriculture and life sciences, providing potential solutions for achieving stable and increased crop yields as well as green and sustainable agricultural development.^[^ ^1^ ^]^ Previous studies have revealed that some nanomaterials play a promotive role in the growth, development, and nutrient absorption of crops, leading to improvements in germination rate, root length, stem length, biomass, and fruit quality.^[^ ^2^ ^]^ The promotive effects of nanomaterials on plant growth involve various pathways, including but not limited to: promoting the absorption of key nutrients, regulating plant water balance, enhancing photosynthetic activity, activating plant hormone synthesis or signaling pathways, boosting antioxidant capacity, and activating plant defense mechanisms.^[^ ^3^ ^]^ Additionally, nanomaterials can also influence the rhizosphere environment and growth media of crops, thus improving the rhizosphere microecological environment and indirectly regulating plant growth.^[^ ^4^ ^]^ The impact of nanomaterials on plants is influenced by various factors, including material type, particle size, surface charge, plant species, and environment.^[^ ^5^ ^]^ Previous studies have indicated that nanomaterials can directly interact with the surface of plant tissues and can enter the plants through both symplastic and apoplastic pathways from the roots, eliciting physiological responses in plants.^[^ ^6^ ^]^ For example, fullerene C70 can be absorbed by rice roots, and then transferred to leaves and seeds. Scanning electron microscope (SEM) observations have shown that Al_2_O_3_ nanoparticles can enter lettuce plants through the roots,^[^ ^7^ ^]^ whereas carbon nanotubes (CNTs) are just adsorbed on the root surfaces of alfalfa and wheat, and are not transported into the plant body.^[^ ^8^ ^]^ However, research on the mechanisms of nanomaterials within plant cells remains relatively limited. Therefore, studying the absorption and transport patterns of nanomaterials can contribute to a better understanding of how nanomaterials regulate plant growth.

Based on their chemical composition, NMs can be categorized into carbon‐based NMs, metal NMs, polymeric NMs and nanocomposites. Carbon nanomaterials have risen to prominence in promoting nutrient absorption and plant growth due to their superior performance, including environmental friendliness, high bioactivity, and relatively low cost compared to other categories.^[^ ^9^ ^]^ Carbon nanosol (CNS) is one of novel carbon nanomaterials produced by pulse electrodeposition of graphite.^[^ ^10^ ^]^ Previous studies have demonstrated the promising application potential of CNS in regulating nutrient absorption,^[^ ^10^, ^11^ ^]^ promoting plant growth,^[^ ^12^ ^]^ and inducing broad‐spectrum disease resistance.^[^ ^13^ ^]^ However, the molecular mechanism underlying the promotive effect of CNS is still unclear,^[^ ^14^ ^]^ and further research is also needed to determine whether its impact occurs on the surface or within the plant body. It remains unclear how plants perceive signals from CNS, and the specific cellular types, genes, and signaling pathways through which CNS promotes plant growth. Notably, the study of transcriptional responses in plants to external stimuli has now entered the single‐cell era.^[^ ^15^ ^]^ Single cell RNA sequencing (scRNA‐seq) can yield high‐resolution cell‐type–specific expression signatures, which reveal new cell types and the developmental trajectories of cell lineages. scRNA‐seq analysis has revolutionized our understanding of plant responses to external stimuli by enabling the examination of gene expression at the resolution of individual cells.^[^ ^16^ ^]^ Single‐cell sequencing technology has enabled to provide novel insight for demonstrating the interaction between nanomaterials and plants. Therefore, constructing plant single‐cell atlases responsive to nanomaterials is of great significance for accurately identifying key targets responsive to nanomaterial and revealing the response patterns of different cell types to nanomaterials.

Water is essential for plant growth and plays a crucial role in the development and maintenance of plant morphology. In addition to free diffusion, water transport within plants largely depends on the expression and activity of aquaporins (AQPs).^[^ ^17^ ^]^ Aquaporins are water transport channels on plasmalemma that play an important role in regulating the water balance of plant cells and tissues.^[^ ^18^ ^]^ Aquaporins are also involved in gas exchange, such as carbon dioxide and nitric oxide, as well as small molecular (urea, lactate, formamide, glycerol, etc.), and the absorption of plant nutrients (such as silicon and boron). According to subcellular localization, the AQP gene family can be divided into five subfamilies: PIPs, TIPs, NIPs, SIPs, and XIPs, among which PIPs and TIPs are mainly expressed on the plasma membrane and tonoplast, respectively.^[^ ^19^ ^]^ AQPs participate in various physiological processes related to water transport and plant growth, such as cell expansion, seed germination, root growth, hypocotyl elongation, and fruit development.^[^ ^19^ ^]^ For instance, the wheat (*Triticum aestivum*) aquaporin *TaPIP2;10* is phosphorylated to enhance photosynthesis, productivity, and innate immunity against pathogens and aphids.^[^ ^20^ ^]^ Similarly, in maize (*Zea mays*), *PIP2;5* influences water relations and growth, with higher hydraulic conductivity of the cortex cells of roots observed in *PIP2;5* overexpression lines and lower conductivity in knockout lines compared to wild‐type plants.^[^ ^21^ ^]^ Several studies have reported that nanomaterials can significantly influence the expression of aquaporin genes in plants. For example, multi‐walled carbon nanotubes activated the expression of the aquaporin gene (*LeAqp1*), thereby promoting seed germination and water absorption by roots.^[^ ^22^ ^]^ Lahiani et al. also confirmed that CNTs increased the expression of aquaporin genes in seeds, thereby promoting seed germination.^[^ ^23^ ^]^ Under cobalt stress, the application of fullerene also increased the expression of aquaporin genes *NIP1‐1* and *TIP2‐1* in wheat.^[^ ^24^ ^]^ However, it is still unclear whether AQPs correlate with CNS mediated growth promotion. Further research on the cell type response patterns of AQPs to nanomaterials is also urgent needed.

Here, we combined single‐cell RNA sequencing (scRNA‐seq) and live‐cell imaging of fluorescent labeling CNS to investigate plant absorption and cellular responses to CNS. To decipher the intricate CNS‐driven plant growth and development regulation in a cell type‐specific manner, we established a single‐cell transcriptome atlas of tobacco (*Nicotiana tabacum*) roots treated with CNS. Significant transcriptomic changes were observed in distinct cell types, particularly in the cortex, pericycle, and trichoblast. Furthermore, trajectory analysis unveiled CNS facilitated the transformation of cellular state. By exploring the cell type‐specific differentially expressed genes (DEGs) between control and CNS‐treated sample and co‐expression network analysis, it was found that CNS specifically activated the expression of multiple PIPs and TIPs subfamily members in epidermal and cortical cells. Further genetic and physiological findings revealed that *tip2;1* and *tip1;1* mutant reduced the CNS‐mediated growth development of plants, suggesting that CNS promoted plant growth by stimulating expression of key aquaporin proteins. Collectively, these results sheds light on the complex regulation of plant growth by nanomaterials, providing a theoretical basis for improving crop yield and quality.

## 2 Results

### 2.1 CNS Promotes Tobacco Growth by Infiltrating into Roots

**Figure 1:**
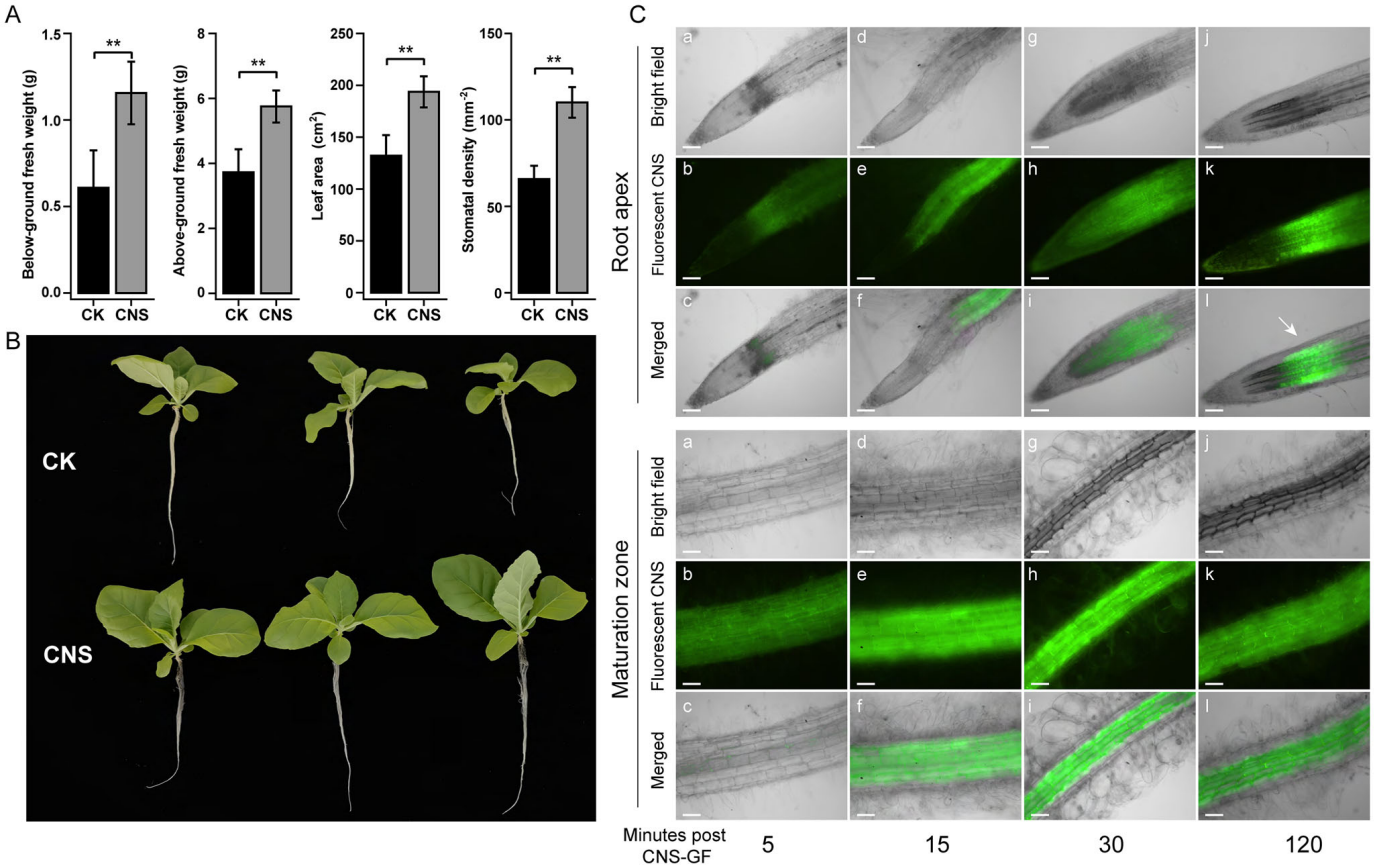
Uptake of CNS and its growth promotion on tobacco. A) Growth parameters of tobacco seedlings after 16 days of 10 mg L^−1^ CNS treatment, including fresh weight of above‐ground and below‐ground parts, leaf area, and stomatal density. Statistically significant differences were calculated by the T‐test (** indicates *P <* 0.01), when compared with control (CK). B) Phenotypes of control and CNS‐treated tobacco plants. C) Confocal microscopy images showing the distribution of fluorescently labeled CNS in the root tip (root cap, meristematic zone, and elongation zone) and maturation zone during fluorescently labeled CNS (green fluorescence) in roots incubated for 5, 15, 30 min, and 2 h. Accumulation of FCNS was observed in the elongation region (indicated by white arrow). Scale bar = 50 µm.

Under hydroponic conditions with CNS treatment, tobacco seedlings exhibited significant growth promotion. Following treatment with 10 mg L^−1^ CNS for 16 days, tobacco seedlings showed markedly improved growth compared to the control samples, with significant increases in both above‐ground and root growth (*P* < 0.05) (**Figure** **1**A,B). The fresh weight of both above‐ground and below‐ground parts of tobacco increased by 59.51% compared to the control (Figure 1A; Table S1, Supporting Information). Stomatal regulation plays a critical role in determining plant growth, development, and yield by controlling photosynthetic activity. The adaxial surface of CNS‐treated leaves exhibited a significantly higher stomatal density (193.69 mm⁻^2^) compared to the control (132.05 mm⁻^2^) (Figure 1A; Figure S1A and Table S1, Supporting Information), which was closely linked to key determinants of photosynthesis and transpiration rates. However, the ultrastructural characteristics of stomata, such as stomatal length, stomatal width, and pore length, showed little or no change between wild type and CNS‐treated plants (Figure S1B,C, Supporting Information). Furthermore, physiological analysis revealed the efficacy of CNS in inducing antioxidant and oxidative stress‐related enzymes in plants, such as ascorbate peroxidase (APX), phenylalanine ammonia‐lyase (PAL), polyphenol oxidase (PPO), glutathione reductase (GR), peroxidase (POD), catalase (CAT), glutathione (GSH), and superoxide dismutase (SOD), whose activities were generally enhanced in the roots (Figure S2 and Table S2, Supporting Information). Furthermore, endogenous levels of abscisic acid (ABA) and indole‐3‐acetic acid (IAA) were significantly elevated in the roots of CNS‐treated plants (Figure S3, Supporting Information). These findings suggest that 10 mg L^−1^ CNS can induce a rise in hormone levels and modulate the activity of antioxidant enzymes, which help scavenge excess reactive oxygen species (ROS), thereby maintaining ROS homeostasis in tobacco.

To assess whether CNS infiltrated into the plant body, fluorescein‐labeled carbon nanosol (FCNS) was used to visualize the spatiotemporal dynamics pattern of CNS absorption and transport in roots. Seedlings were cultured in green fluorescent‐labeled carbon nanosol, and the entry and distribution of nanoparticles was tracked using laser confocal microscopy. It was observed that CNS could enter tobacco roots and accumulated at the root tip (Figure 1C). After exposure to FCNS for 5 min, minimal green fluorescence was attached to the root surface as well as in the intercellular space. At 15 min, FCNS accumulation appeared in the epidermal cell walls and mature vascular bundles, and after 30 min, green fluorescence signals emerged in the elongation zone of the root tip, indicating the initiation of FCNS penetration into the roots. After 2 h of treatment, green fluorescence was primarily present in the mature zone and elongation zone, with concentrating in the cortex and vascular cells near the elongation zone (as indicated by arrows in Figure 1C). Additionally, relatively little FCNS absorption and accumulation were observed in the root cap, while the fluorescence intensity in the trichoblast and the root meristem remained low. Although the whole root was exposed to FCNS, the fluorescence always appeared on the upper parts of root tips rather than the meristem during treatment period. These results suggested that CNS initially adhered to the root surface and penetrated the root tissues within half an hour. CNS could penetrate the cell wall and plasma membrane to reach the vascular bundle cells, indicating its potential transportation pathways including both apoplastic and symplastic pathways. To complement the light microscopy results, we exploited the high electron density of gold‐based materials to directly visualize CNS in plants with transmission electron microscopy (TEM), revealing that CNS were also observed localize within the intracellular spaces (Figure S4, Supporting Information).

### 2.2 Construction of Single‐Cell Transcriptomic Atlas of Tobacco Roots Responsive to CNS treatment

Due to the uneven distribution of CNS in different cell types of roots, a single‐cell transcriptomic atlas of tobacco roots responsive to CNS was constructed to explore the molecular mechanisms underlying CNS‐regulated plant growth at single‐cell resolution. Here, we apply scRNA‐seq to tobacco to capture gene expression in different root cells. Whole tobacco roots from 16 d CNS treated and control seedlings were used to generate protoplasts for transcriptome analysis using the 10X Genomics platform. After filtering duplicates, low‐quality cells and genes, two groups under CNS and control conditions captured a total of 4015 and 3882 cells, respectively (Table S3, Supporting Information). On average, each cell detected 2359 unique molecular identifiers (UMIs). These UMIs corresponded to the expression of a median of 1433 genes per cell and a total of 35731 genes, accounting for 53.5% of the total genes of *N. tabacum* SR1 genome.

**Figure 2:**
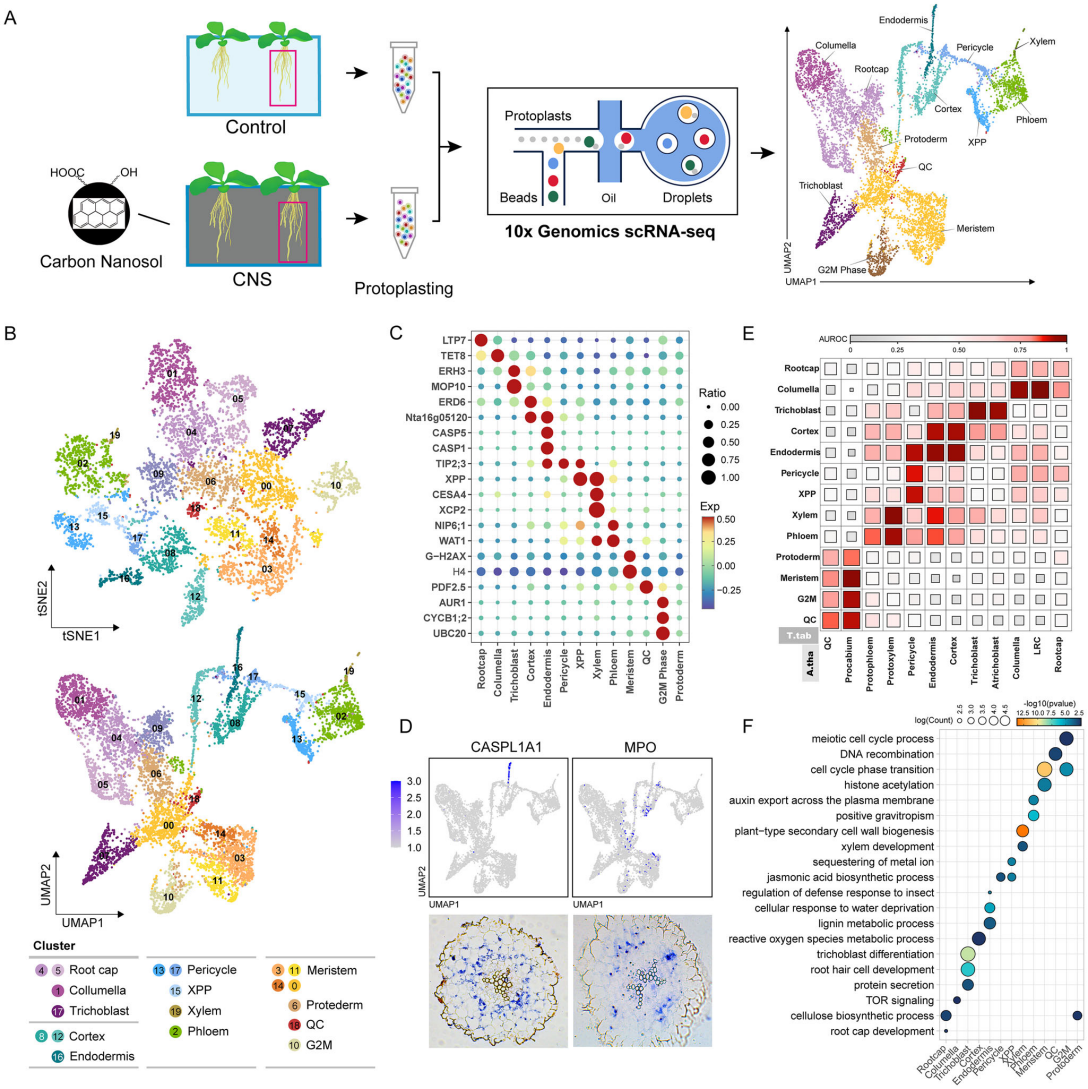
Single‐cell transcriptome atlas of tobacco root responsive to CNS. A) Overview of tobacco root scRNA‐seq workflow. Protoplasts were isolated from root of control and CNS‐treated plants, respectively. B) t‐SNE and UMAP visualization of 19 cell clusters of tobacco roots. C) Dot plots showing expression of representative marker genes in each cell type. Cell types are indicated on the x‐axis. Colors denote corresponding expression levels. D) RNA in situ hybridization and UMAP visualization of expression patterns of two marker genes, including *CASPL1A1* in endodermis, and *MPO* in cortex. E) Heatmap showing correlation between tobacco and Arabidopsis root single‐cell transcriptomes. F) Representative GO enrichment analysis of the cell type‐enriched genes.

Cells were projected onto two dimensions using the Uniform Manifold Approximation and Projection (UMAP) and t‐distributed stochastic neighbor embedding (t‐SNE) method. An unsupervised analysis grouped tobacco root cells into 19 clusters (**Figure** **2**A,B). The previously reported marker genes were collected to annotate the cell types of each cluster (Figure 2C; and Table S4, Supporting Information). The largest cell clusters (cluster 0) were annotated as meristematic cells due to predominantly expressed marker genes like the Histone H4 and G‐H2AX (Figure 2C; Figure S5A, Supporting Information). The expression distribution of *Cellulose Synthase A4* (*CESA4*) and *Xylem Pole Pericycle* (*XPP*) was used to determine the pericycle (cluster 15 and 17). Cell clusters 2 and 19 with high expression levels of *Walls Are Thin 1* (*WAT1*) were assigned as vasculature and further classified as xylem and phloem based on the specific expression patterns of *Xylem Cysteine Protease 2* (*XCP2*) and *Nodulin 26‐like Intrinsic Protein 6;1* (*NIP6;1*), respectively. Additionally, the *Casparian Strip Domain Protein 5* (*CASP5*) and *Casparian Strip Domain Protein 1* (*CASP1*) involved in casparian strip were expressed specifically in endodermis (cluster 16). Clusters 8 and 12 were annotated as cortex cells based on the expression pattern of *Early Responsive to Dehydration 6* (*ERD6*). Meanwhile, the *Early Response to Heat Shock 3* (*ERH3*) and *Modifier of SNC1.10* (*MOP10*) were differentially expressed in trichoblast cells (cluster 17) (Figure 2C). Quiescent center cells showed a unique expression profile of its marker gene *Plant Defensin 2.5* (*PDF2.5*) in cluster 16. Considering the high expression of cell cycle‐related genes such as *Auxin‐responsive protein 1* (*AUR1*), *Cyclin B1;2* (*CYCB1;2*), and Ubiquitin‐conjugating enzyme 20 (*UBC20*) in cluster 10, it was designated as the G2 M phase cell population (Figure S5B,C, Supporting Information).

To validate the accuracy of cell type assignment, in situ hybridization experiments were conducted for endodermal marker gene *Casparian Strip Lignin A1* (*CASPL1A1*) and cortical marker gene *Myeloperoxidase* (*MPO*), confirming the assignment of cortex and endodermis cell identities and validating the accuracy of cell type annotation in this study (Figure 2D). Based on marker genes, we were unable to assign a specific cell type identity to cell cluster 6. However, using interspecies correlation comparison between tobacco and *Arabidopsis* (Figure 2E), cell cluster 6 was found to be in an intermediate state between epidermal cells and meristematic cells and was therefore designated as protoderm.

Through gene ontology (GO) enrichment analysis of differentially cell types‐specifically expressed genes (Table S5, Supporting Information), we further evaluated the credibility of cell type classification. The results indicated that the majority of enriched functions or pathways were consistent with the differentiation or specific physiological functions of the respective cell types (Figure 2F). For instance, genes specifically expressed in meristematic tissue were enriched in cell cycle‐related pathways, while those in xylem cells were associated with secondary cell wall biosynthesis and xylem development. Additionally, genes specific to endodermal cells were related to responses to water deprivation and lignin metabolism pathways, whereas those in trichoblast cells were enriched in pathways involved in trichoblast differentiation and protein secretion. In summary, this study constructed a single‐cell transcriptomic atlas covering the major cell types in tobacco roots and captured the changes in gene expression in response to CNS at the cellular level.

### 2.3 Cell‐Type‐Specific Response to CNS in Tobacco Roots

**Figure 3:**
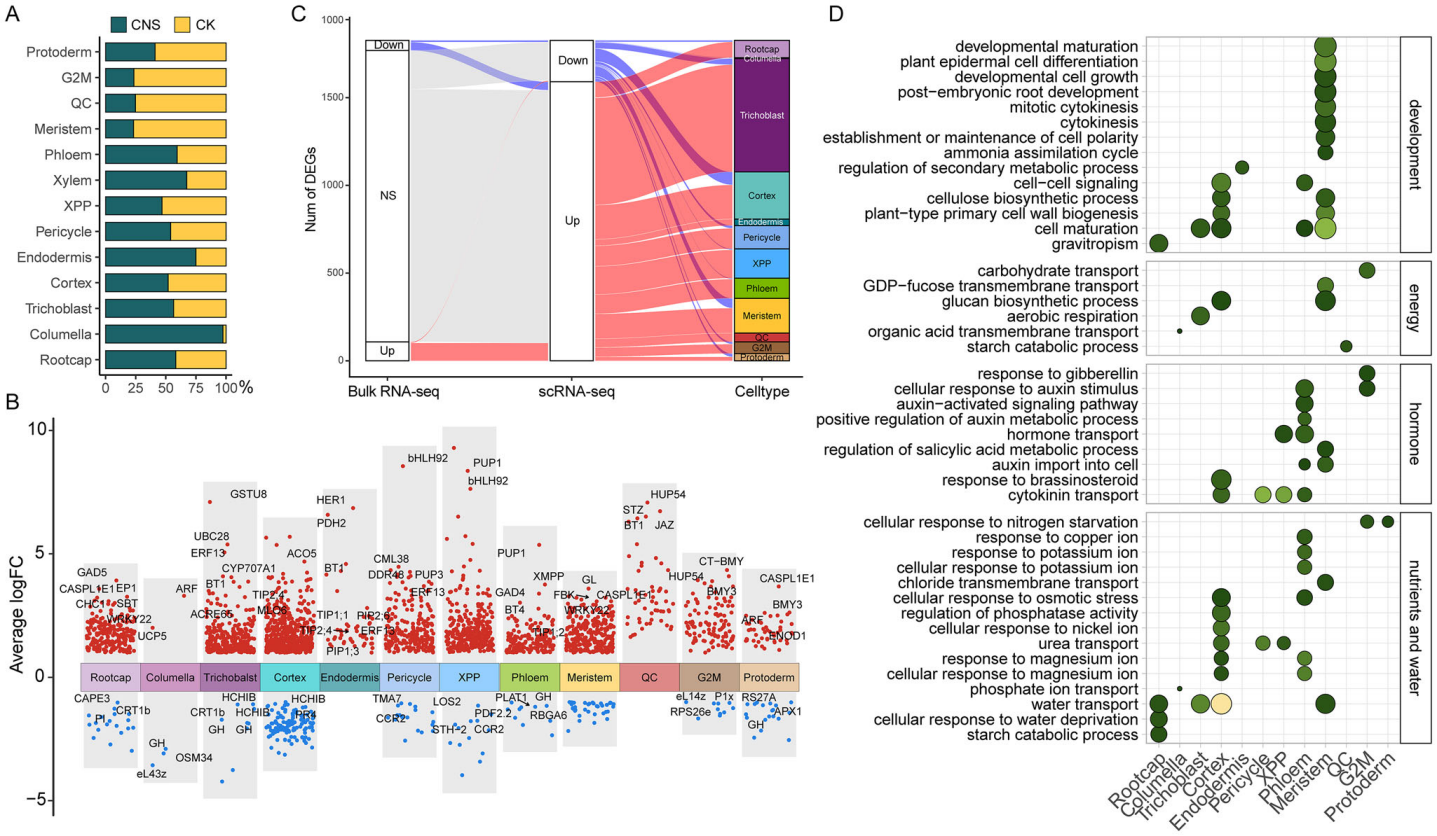
Cell‐type‐specific response of tobacco roots to CNS. A) The proportions of cells from different samples in each cell type. B) Manhattan plot showing differentially expression gene (DEG) between control and CNS‐treated samples in different cell types. C) Sankey diagram showing that DEGs between Bulk RNA‐seq and scRNA‐seq. D) GO enrichment analysis of DEGs for each cell type.

In order to investigate difference between cell type response to CNS in tobacco roots, cell proportions comparison and DEGs between CNS‐treated and control samples were conducted based on the single‐cell atlas (Table S6, Supporting Information). Landscapes of transcriptome atlases made by cell populations from different samples exhibited alterations in distinct cell clusters (Figure S5D, Supporting Information). For instance, the cell types related to the meristematic tissues (including the quiescent center, meristem cells, and G2M phase cells) were mainly composed of cells from the control samples (**Figure** **3**A), while the columella, endodermis, and xylem cell clusters were predominantly composed of cells from the CNS‐treated samples.

By comparing gene expression profile between CNS and control samples in the same cell type, a total of 1210 DEGs whose expression was altered by CNS treatment, were identified (Figure 3B). The DEGs in epidermis (309 genes), cortex (594 genes), and phloem (294 genes) were the largest, reflecting those cell types are more sensitive to CNS treatment. In particular, the ethylene response factor *ERF13*, which was involved in regulating root growth,^[^ ^25^ ^]^ was significantly increased in vascular, cortical, and root hair cells upon CNS treatment. Additionally, the expression level of *bHLH92*, playing important roles in the context of abiotic stress physiology and hormone response,^[^ ^26^ ^]^ was markedly elevated in the pericycle under CNS treatment. It was noteworthy that enhanced expression of multiple aquaporin genes (*TIP2;1*, *TIP1;1*, *TIP2;5*, *PIP2;4*, *PIP1;5*, etc.) was observed in cortex, endodermis, and trichoblast cells. Moreover, when we compared gene expression between traditional bulk RNA‐seq and single‐cell atlas, the expression levels of most differentially expressed genes identified by single‐cell dataset showed no significant differences in bulk RNA‐seq (Figure 3C), indicating that these genes responded to CNS in a cell‐type‐specific manner, which was masked by mixed cell types during conventional bulk RNA‐seq analysis. Thus, it highlighted the importance of single‐cell resolution analysis in capturing the heterogeneity of gene expression in response to CNS.

GO enrichment analysis for these DEGs in each cell type revealed distinct functions in their respective cell types (Figure 3D). For example, CNS‐responsive pathways related to cell differentiation and the cell cycle were significantly enriched in meristem. Genes responsive to CNS in vascular bundle (xylem, phloem, pericycle) cells were enriched in hormone transport and signal transduction pathways, including auxin and gibberellin. CNS also enhanced the expression of pathways related to photosynthate transport and energy metabolism in meristematic tissue and cortex cells. Furthermore, CNS treatment promoted the absorption and transport of nutrient ions (N, K, P, and Cu) and small molecules such as urea in cortex and vascular bundle cells. Further genetic and phenotype observation for nitrate transporter 3.1 (*nrt3.1*) and potassium transporter 8 (*pot8*) mutants in tobacco (Figure S6, Supporting Information), indicated that the promotion of CNS‐induced growth was significantly reduced by key nutrient transporters. Additionally, genes involved in water transport and response to dehydration were significantly enriched in epidermal and cortical cells. These results suggested that epidermial, cortical and meristematic tissue, especially the cortex and trichoblast, were the major regions that responded to CNS treatment.

### 2.4 Cell‐Type‐Specific Co‐Expression Regulatory Network Responsive to CNS

**Figure 4:**
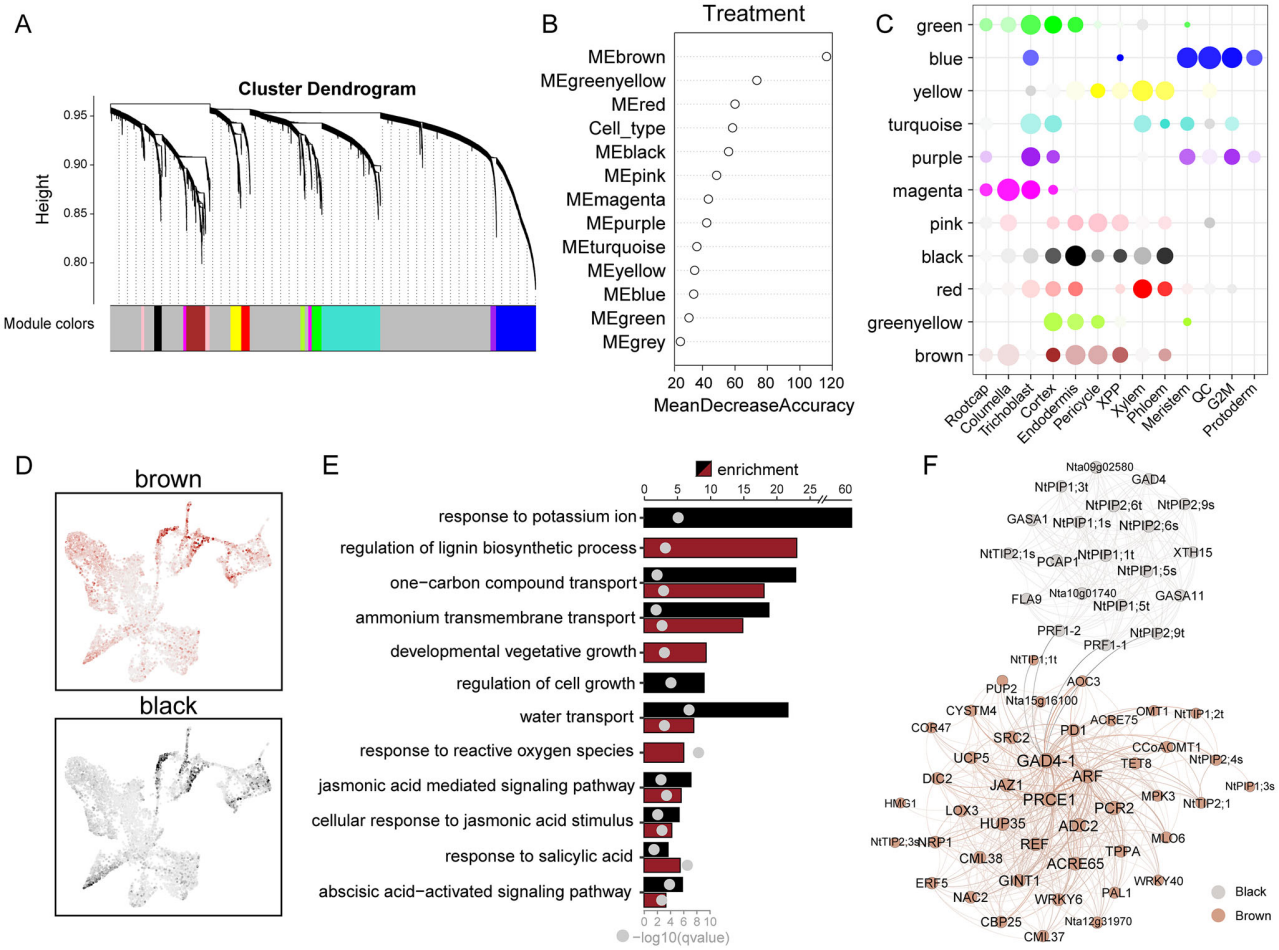
Cell‐type‐specific co‐expression regulatory network responsive to CNS treatment. A) Clustering of multiple co‐expressed genes modules. The color bar beneath the dendrogram represents the module assignment of each gene. B) Accuracy of cell type classification based on the random forest model. C) Expression patterns of different gene modules among different cell types. D) UMAP heatmap displaying expression patterns of brown and black gene modules across different cell types. E) GO enrichment analysis of genes in brown and black modules. F) Visualization of hub genes and network connections in brown and black modules.

To further explore the impact of CNS on gene expression regulation at single cell level, a co‐expression network analysis was performed using hdWGCNA. The identified co‐expression network was composed of 11 modules harboring 75 to 1035 genes, and one unassigned module (gray) (**Figure** **4**A; and Table S7, Supporting Information). By employing the random forest algorithm, we identified key modules with distinguishing features between control and CNS treated samples. Notably, the brown, greenyellow, red, black, and pink modules exhibited the highest classification accuracy, with the brown module significantly involved in cell classification, potentially related to CNS response (Figure 4B). The black and brown modules were specifically expressed in both cortex and vascular bundle cells, with the highest expression levels observed in the cortex and endodermis (Figure 4C,D), consistent with the observed specific accumulation of CNS in the root system, suggesting their potential significant regulation by CNS.

GO enrichment analysis revealed that genes enriched in black and brown modules were mainly associated with processes such as phospholipid transport, water and carbohydrate transport, lignin biosynthesis, and nutritional growth (Figure 4E). Remarkably, the black module contained 11 genes encoding aquaporins, while the brown module included 7 AQPs. A co‐expression network was constructed using hub genes from the brown and black modules (Figure 4F). Among this network, key genes such as *PRCE1* (*PSI‐INTERACTING ROOT‐CELL ENRICHED 1*) involved in phosphate starvation response, *GAD4* encoding glutamate decarboxylase, *PCR2* (*PLANT CADMIUM RESISTANCE 2*) implicated in cadmium stress resistance, *XTH15* involved in cell wall formation, and genes related to phenylpropanoid metabolism like *CCoAOMT1* and *ADC2*, were found to be co‐expressed with multiple aquaporin genes (PIPs and TIPs). Transcription factors including *WRKY6*, *WRKY40*, *ERF5*, and *NAC2* were also identified, potentially participating in transcriptional regulation of plant responses to CNS. These findings provided insights into the regulatory networks of different cell types in tobacco roots responding to CNS treatment at the cellular level.

### 2.5 CNS Accelerated the Differentiation Trajectory of Root Cells

**Figure 5:**
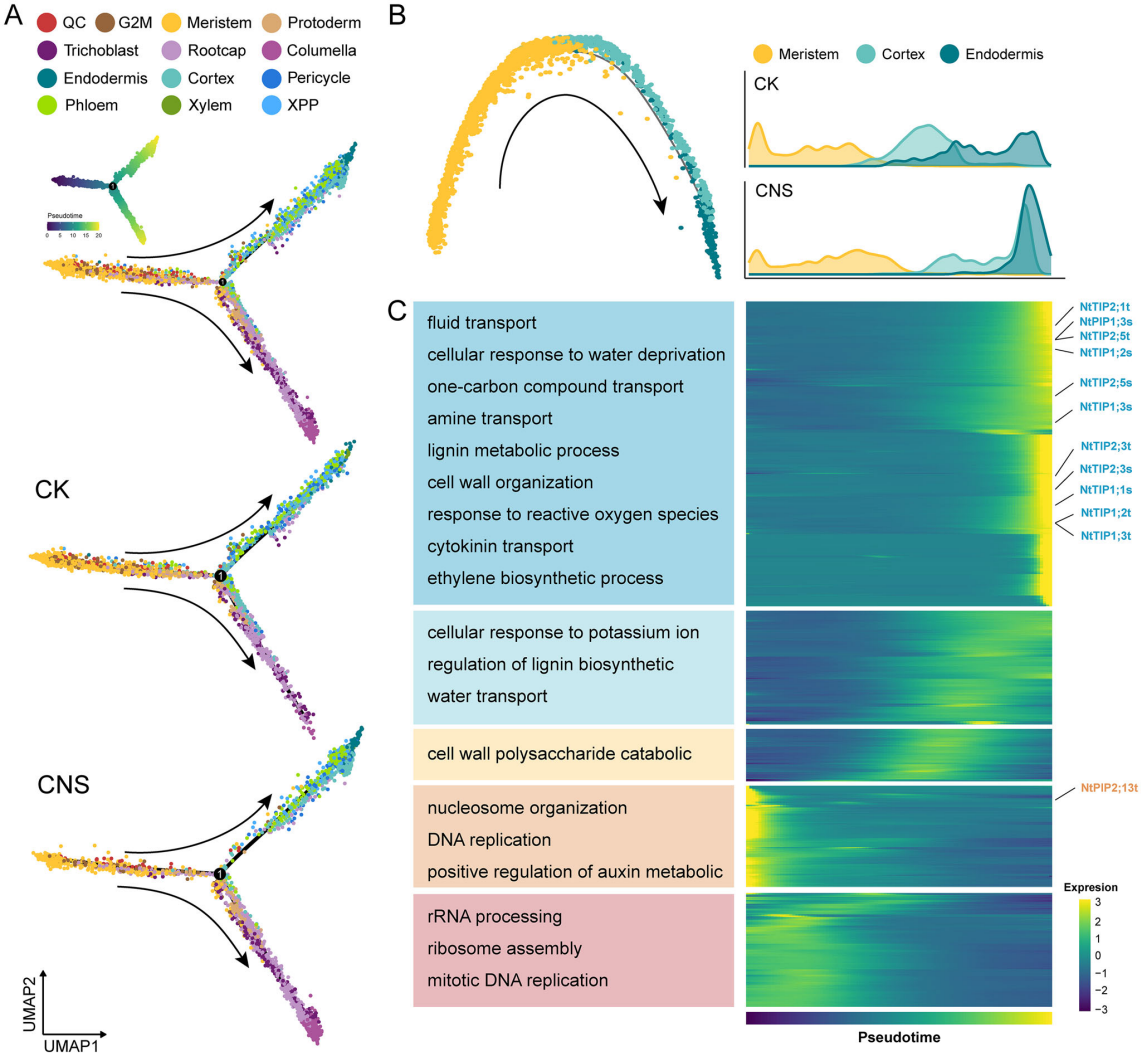
Differentiation trajectory of control and CNS‐treated root cells. A) Pseudotime analysis of all cells of control and CNS treated samples. Each dot indicates a single cell, and the color of the upper right corner represents different cell types. B) Differentiated trajectories of cortex and endodermis. C) Heatmap showing the expression of differentially expressed genes for the cortical cells over pseudotime. Color bar indicates the relative expression level.

To further understand how CNS changed the cell fate transitions during root development, we carried out trajectory analysis using monocle2. A trajectory curve for all cell types was then generated (**Figure** **5**A). The developmental trajectory for tobacco roots could be delineated into three distinct branches, originating from highly proliferative cells and branching into cortical and vascular bundle cells, as well as epidermal cells (including root cap and trichoblast cells). The root cells treated with CNS appeared to exhibit a higher degree of differentiation, with more cell populations developed at the higher pseudotime time. To dissect the developmental dynamics of the CNS‐induced cell type, we focused on the cortical and epidermial cells, placing them in “pseudotime” order along a trajectory that described their maturity state (Figure 5B; Figure S7A, Supporting Information).

The percentage of mature cortex, endodermis, and trichoblast cells was significantly elevated in the CNS‐treated sample relative to the control sample, while the proportion of undifferentiated cells was lower (Figure 5B; Figure S7A, Supporting Information). Cells of cortex and endodermis were more likely to differentiate into mature state upon CNS treatment, suggesting these cells might have more plasticity during response to CNS treatment. These results suggested that CNS treatment had the potential to drive both global and cell‐specific transcriptomic reprogramming in the roots, indicating a potential mechanism for promoting cell differentiation, especially on cortex, endodermis, and trichoblast cell types.

Given the observed trend in cell population shifts from control to CNS‐treated samples, we characterized gene expression changes during root cortical and epidermial development (Figure 5C; Figure S7B and Table S8, Supporting Information). During the maturation process of root hairs and cortical cells, many GO terms were associated with the transport of nutrients and water pathways. Additionally, some genes related to lignin metabolism and cell wall formation were also enriched. As the pseudotime increases, GO terms associated with stress and stimulus response were gradually emerging. It was noted that a clear gradual increasing pattern for TIPs and PIPs gene was observed during trichoblast, cortex, and endodermis differentiation process, implying their important roles during CNS treatment.

### 2.6 Cell‐Type‐Specific Response to CNS was Mediated by Aquaporin Protein Family

**Figure 6:**
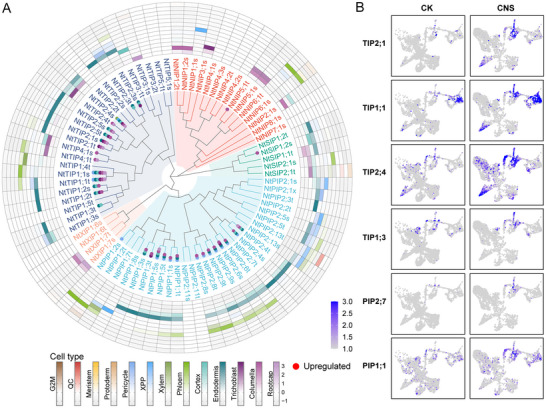
Cell‐type‐specific response of tobacco aquaporin protein family to CNS treatment. A) Gene expression data for aquaporin proteins using control and CNS‐treated samples in 13 major cell types. The maximum‐likelihood phylogeny was built using protein sequence alignment of the AQPs. Undetectable levels were shown as blank. Each protein was colored‐coded for sub‐classes as NIP (red), PIP (light blue), TIP (navy), SIP (green), and XIP (orange). Dot point with color indicated significant increased expression in corresponding cell type compare to control. The heatmap showed relative expression levels normalized for each gene. B) UMAP heatmap depicting the cellular‐specific response of TIPs and PIPs to CNS.

Considering the significant differential expression of aquaporin proteins across different cell types and important roles in co‐expression regulatory networks, we systematically investigated the expression patterns of tobacco aquaporin genes (*NtAQPs*) family in different cell types. In the tobacco NtaSR1 genome, 78 NtAQP genes were identified, consisting of 29 PIPs, 25 TIPs, 15 NIPs, 5 SIPs, and 4 XIPs (**Figure** **6**A; and Table S9, Supporting Information). By comparing the expression patterns of NtAQP genes between different cell types, significant differences among different subfamilies across root cell types were observed. As shown in Figure 6A, NIP and XIP subfamilies exhibited higher expression in the root cap, while PIP and TIP subfamilies showed higher expression in the cortex and endodermis cells, with a few members also specifically expressed in the pericycle or vascular sheath. The diversity of aquaporin protein expression in root cells suggested their potentially differentiated roles in transmembrane water transport during CNS treatment.

Interestingly, a substantial increase in genes encoding aquaporins, involved in water transport, was observed in multiple cell types following CNS treatment. 33 NtAQP genes were markedly induced by CNS treatment in at least one cell type (Figure 6A; and Table S10, Supporting Information). After exposure to CNS, PIPs were upregulated in root cap, endodermis, and vasculature, such as *NtPIP2;4*, *NtPIP2;6*, *NtPIP1;5*, and *NtPIP1;3* (Figure 6B). Similarly, CNS treatment activated the expression of the most members of TIPs in epidermal cells (including root cap, columella, and trichoblast) and cortical cells (including cortex and endodermis). For example, the expression of *NtTIP2;1*, *NtTIP1;1*, *NtTIP2;4*, and *NtTIP1;3* dramatically increased in these cell types (Figure 6B). In addition, increased expression levels were also observed in root cap cells for a few members of NIPs and SIPs subfamilies (*NtNIP5;1* and *NtSIP1;2*). Few *NtAQPs*, such as *NtPIP2;6*, *NtPIP2;8*, *NtPIP2;9*, were expressed in the pericycle and xylem, while *NtAQPs* were almost not affected by CNS in meristematic cells. These results indicated that multiple aquaporin protein family members might mediate the cellular‐specific response to CNS.

### 2.7 AQPs were a Key Factor Involved in CNS‐Mediated Promotion Growth of Plants

**Figure 7:**
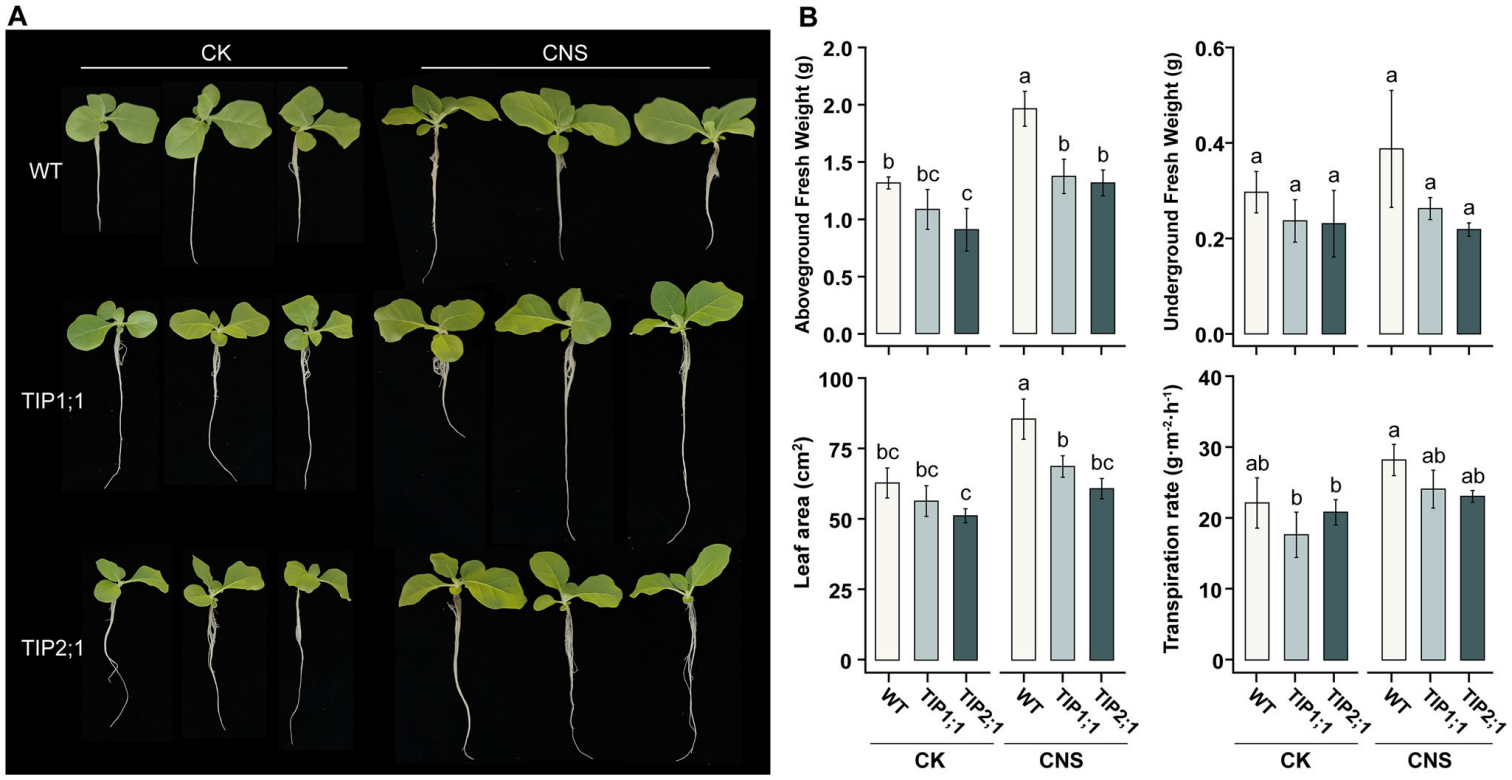
The growth promotion effect of CNS for tobacco on different AQPs mutant (*TIP1;1* and *TIP2;1*). A) The phenotype of leaves and roots of the tobacco seedlings (WT: wild type, *TIP1;1*: *NtTIP1;1* RNAi line, *TIP2;1*: *NtTIP2;1* RNAi line) were grown in a hydroponic solution with 10 mg L^−1^ CNS for 10 days. B) The biomass (aboveground and underground fresh weight), leaf area and transpiration rate were measured when the tobacco plants were grown under CNS for 10 days. Bars represent means ± SD from 5 replicates. Significant differences were assessed using one‐way analysis of variance (ANOVA) with Tukey's HSD test. Different letters indicate statistically significant differences between samples (*p* ≤ 0.05).

Previous results have demonstrated that CNS could stimulate plant growth and activate the expression of tonoplast intrinsic protein *TIP2;1* and *TIP1;1* (Figure 6B). To verify the roles of TIP genes in CNS‐induced plant growth, RNA interference (RNAi) mutants for two significant members (*tip2;1* and *tip1;1*) were constructed for growth promotion effect comparison between wild type and CNS treatment. The biomass of the *tip2;1* and *tip1;1* mutant was significantly lower than wild‐type plants in normal condition, showing reductions of 30.77% and 17.69%, respectively (**Figure** **7**A,B; and Table S11, Supporting Information). Additionally, a slight decrease in transpiration rate was observed in the mutants (Figure 7B; and Table S11, Supporting Information). As for wild type, there was a notable improvement in vegetative growth in roots and shoots under CNS treatment. However, these growth promotion effects were significantly weakened in *tip2;1* and *tip1;1* mutants, suggesting that these genes might play important roles during CNS treatment. Specifically, after CNS treatment, the biomass of the *tip1;1* and *tip2;1* mutants were increased by 25.3% and 36.28%, respectively, which was significantly lower than the 45.62% improvement observed in wild type plants (Figure 7). Overall, these results indicate that *TIP2;1* and *TIP1;1* might play important roles in mediating the growth‐enhancing effects of CNS in plants.

## 3 Discussion

### 3.1 Carbon Nanosol Exhibited Distinct Accumulation in the Root of Plant

A comprehensive understanding of plant‐nanomaterial interactions first requires clarification of whether nanomaterials enter the plant body. However, systematic studies on the absorption and transport of nanomaterials in plants are still lacking. In this study, we tried to utilize fluorescent labeling and laser confocal microscopy to observe and track the absorption and transport of carbon nanosol (CNS) in tobacco roots, clarifying ability of CNS to penetrate the cell wall and membrane to promote tobacco growth. The absorption and transport of NMs in plants depend on the physiological and structural characteristics of plant cells, interactions between nanomaterials and the environment, and the properties and stability of NMs.^[^ ^35^ ^]^ Due to the presence of biological barriers (cell walls and membranes) of plants, the entry and transport of nanomaterials are restricted. NMs with diameters smaller than the pores of cell wall can enter the plant roots via the apoplastic pathway.^[^ ^36^ ^]^ It has been reported that NMs with diameters ranging from 40–50 nm can penetrate the cell wall.^[^ ^37^ ^]^ Moreover, NMs can enlarge pores and induce the formation of new pores of cell wall, which may increase their absorption. Furthermore, NMs can enter the cytoplasm through endocytosis, via carrier proteins or ion channels.^[^ ^37^ ^]^ With an average size of ≈30 nm and a negative charge, CNS may penetrate the cell wall and enter the plant cell due to the abundance of hydroxyl and carboxyl functional groups on its surface. Nanoparticles accumulated in plant roots can be further transported to different tissues in the aboveground parts of plants, such as stems, shoots, and even seeds. For example, previous studies have shown that gold nanoparticles can accumulate in the stems of rice plants.^[^ ^38^ ^]^ Fullerene C70 absorbed by rice roots can be transported to the shoots, and the transport of fullerene is synchronized with the absorption of water and nutrients.^[^ ^39^ ^]^ More studies are still needed to clarify whether CNS is further transported to other organs via the roots in future.

### 3.2 ScRNA‐seq Technology Revealed the Heterogeneous Response of Plant to CNS

Single‐cell omics techniques provide a powerful tool for deepening our understanding of the interaction between nanomaterials and plants at single‐cell resolution. In recent years, they have been applied in plant science, particularly in studying the developmental processes of different organs, stress responses, and plant‐microbe interactions.^[^ ^33^, ^34^ ^]^ An important question about plant responses to nanomaterials is whether such responses are nonuniform across different cell types. However, past studies investigating plant‐nanomaterial interactions mainly depend on assays from bulk tissue (i.e., whole leaf or root), and there have been no reports on studies concerning plant responses to nanomaterials in single‐cell mode. In this study, we have successfully profiled the single‐cell transcriptome atlas of CNS‐treated root cells to explore whether CNS alter gene expression in a cell‐type–specific manner. The results of this study demonstrated significant differences among different cell types in the response to CNS. The expression profiles of cell types such as epidermis, cortex, and phloem showed significant responses to CNS treatment. Interestingly, most of the differentially expressed genes identified by single‐cell dataset did not show significant differences in bulk RNA‐seq data, implying that these genes might respond to CNS in a cell‐type‐specific manner. These findings also indicated that single‐cell technology has the potential ability to obtain differential expression information that was masked by cell type mixing in RNA‐seq data, further demonstrating the necessity of applying single‐cell technology.

Subsequently, CNS could induce both global and cell‐specific transcriptomic reprogramming in the roots, indicating a potential mechanism for promoting cell differentiation. Trajectory analysis suggested that root cells treated with CNS trended to be differentiated cell, especially on cortex, endodermis, and trichoblast cell types. These observations suggested that trichoblast and cortical cells might rapidly specialize under CNS treatment, assuring water and nutrient supply from the primary root.

### 3.3 AQPs Played an Important Role in CNS‐Regulated Plant Growth Promotion

In this study, we found that the expression of multiple TIP and PIP aquaporin genes in tobacco roots was broadly induced by CNS treatment, and the mutant of these genes impaired CNS‐mediated plant growth promotion. According to the single‐cell transcriptome atlas constructed in this study, the tobacco AQP family was specifically expressed in epidermal cells (including root cap, trichoblast, and epidermal cells) and cortical cells (including cortex and endodermis). Lignin deposition in the cell walls of endodermal cells formed casparian strips, which created resistance to radial transport through the apoplast. These AQP members might partially compensate for the extracellular barrier of endodermal cells (apoplastic barriers) and promote water flow from the cortex to the vascular bundle. Additionally, water channel proteins distributed on the root surface might facilitate water influx into the cortex. These results were consistent with the reported localization of aquaporins in other plants (rice,^[^ ^27^ ^]^ maize,^[^ ^28^ ^]^ and radish^[^ ^29^ ^]^). Previous evidence have demonstrated that CNTs could promote water absorption and accelerate tomato seed germination by regulating aquaporins in seed coats.^[^ ^30^ ^]^ After exposure to CNS, the expression of aquaporin genes involved in transmembrane water transport in tobacco epidermal and cortical cells were broadly upregulated, indicating that CNS might enhance root water absorption capacity, improve plant water status, and promote plant growth and development. Further genetic and physiological findings revealed that *tip2;1* and *tip1;1* mutants significantly reduced CNS‐induced growth promotion of plants, indicating AQPs were required for plant growth promotion of CNS. Furthermore, the down‐regulation of these TIP aquaporins led to growth inhibition, emphasizing their crucial role in plant development. Based on these findings, aquaporins were thought to play crucial roles in CNS‐induced growth enhanced, although the underlying mechanism was unknown.

However, these findings also raised important questions whether changes of aquaporin expression in specific cell types could totally translate to overall plant hydraulic function. Similar studies on *PIP2;5* in maize revealed that increased hydraulic conductivity could be observed in cortex cells but no significant effect on whole‐root conductivity in overexpression lines, suggesting that alterations in water permeability at the cellular level did not necessarily impact the entire root system's water transport efficiency.^[^ ^21^ ^]^ This might be due to the radial non‐uniformity of plasma membrane permeability and the saturation of AQPs in key tissues, such as the endodermis and exodermis, which possessed apoplastic barriers. This implied that the regulation of overall root hydraulic conductance might be localized to specific cell types. Therefore, in CNS‐treated TIP RNAi mutants (e.g., *tip2;1* and *tip1;1*), plant growth was partially restored, although it was slightly reduced compared to CNS‐treated wild type plants, indicating that other AQPs might compensate for the loss effect of specific members. Thus, the effects of CNS on growth promotion might result from a complex interplay of multiple AQPs rather than changes in a single AQP's expression. Further research was still needed to unravel the regulatory networks controlling AQP activity and their precise role in CNS‐induced plant growth enhancement.

Although our study primarily focuses on the early stages of tobacco growth and the molecular mechanisms involved in CNS‐induced growth promotion, the quality of tobacco leaves, particularly in terms of color and aroma, is largely determined during the maturation and postharvest stages. How CNS treatments influence these key quality traits at later stages is still unknown, which could provide valuable insights into the broader agricultural and industrial applications of CNS in tobacco cultivation. The findings of this study not only advance our understanding of plant‐nanomaterial interactions at the cellular level but also open up new avenues for the application of CNS in agriculture. The ability of CNS to promote plant growth through the regulation of AQPs could have significant implications for improving crop yields, particularly in regions facing water scarcity or soil nutrient limitations. By enhancing water and nutrient uptake via AQP‐mediated pathways, CNS could help crops maintain relative optimal growth under harsh environmental conditions, such as drought or nutrient deficiency. Furthermore, CNS may offer an environmentally friendly alternative to conventional fertilizers and growth enhancers, reducing the need for chemical inputs and their associated environmental impact.

## 4 Conclusion

The successful application of scRNA‐seq technology provided us with an opportunity to understand the plant‐growth‐promotion mechanism of CNS at the single‐cell level. In this study, we visualized CNS absorption and transport in tobacco roots using fluorescence labeling and laser confocal microscopy. The single‐cell transcriptomic atlas of tobacco root tissue exposed to CNS revealed various cell types responsive to CNS, which exhibited strong transcriptional heterogeneity. Based on trajectory analysis, we found that CNS promoted cell developmental trajectory extending and differentiation. Furthermore, the aquaporin family was found to be specifically expressed in epidermal and cortical cells, with many *PIPs* and *TIPs* upregulated under CNS treatment. The essential role of the *TIP2;1* and *TIP1;1* during CNS‐induced growth effect was confirmed by the growth promotion experiment using RNAi mutants. Overall, this study elucidated the transportation pathways of CNS in promoting crop growth and the transcriptional heterogeneity of plant root response to carbon nanosol, providing a reference for further exploration of the rational application of CNS in sustainable agriculture.

## 5 Experimental Section

### Plant Growth and Carbon Nanosol Treatment

Seeds of tobacco (*Nicotiana tabacum*, cv. K326) were surface‐sterilized and grown on 1/2 MS (Murashige and Skoog) solid medium supplemented with 3% sucrose. After germination, the plants were grown for 15 days under a 16 h light/8 h dark cycle at 28 °C.Then, tobacco seedlings were transferred to hydroponic containers with in 1/4 Hoagland solution at the stage of four true leaves. After a three‐day acclimatization period, the seedlings were treated with 10 mg ml^−1^ carbon nanosol, while the control group was maintained in 1/4 Hoagland solution. The tobacco seedlings were grown for 16 days under a 16 h light/8 h dark cycle at 28 °C, and morphological parameters of the plants (fresh weight, plant height, root length, etc.) were measured. Each treatment included six replicates, and samples were taken for transcriptomic and single‐cell transcriptomic sequencing simultaneously.

### Preparation and Characterization of CNS

The carbon nanosol was obtained from Beijing Nais New Materials Technology Co., Ltd., and prepared by pulse electrodeposition of graphite, purity > 99.9%. The carbon nanomaterial exhibited a particulate morphology with a size distribution ranging from 18 to 70 nm and an average particle size of ≈30 nm. Surface analysis confirmed the presence of hydrophilic COOH and OH functional groups, and the CNS displayed the typical feature of sp^2^‐hybridized graphitic carbon.^[^ ^31^ ^]^

### Preparation of Fluorescent Carbon Nanosol and Microscopic Observation

To visualize the transport and accumulation of CNS within plant tissues, 1‐(3‐dimethylaminopropyl)‐3‐ethylcarbodiimide hydrochloride (EDC) was used as an activator for carboxyl groups to modify the surface functional groups of carbon nanosol and attach green fluorescent groups.^[^ ^32^ ^]^ Tobacco seedlings grown on 1/2 MS medium for 30 days were exposed to 100 mg L^−1^ CNS for 5, 10, 30 min, and 2 h, and then rinsed with deionized water to remove fluorescent carbon nanosol from the root surface. Laser confocal microscopy (Leica, Germany) was used to observe the entry of CNS into the root tips, with an excitation wavelength of 488 nm and an emission wavelength of 495–530 nm.

### SEM Observation and Evaluation of Stomatal Micrographs

The adaxial surface of tobacco leaves was observed and photographed in a variable‐pressure SEM (HITACHI UHR FE‐SEM Regulus8100; Hitachi Ltd, Japan) at 400×–100000× magnification, followed by microscopic image processing with image J application (https://Imagej.nih.gov/ij/). To measure stomatal density (SD), the number of stomata was counted and divided by area of field‐of‐view (0.280 mm^2^) to obtain the amounts of stomata per millimeter square of leaf. The width and the length of the stomata were measured at 200‐µm scale.

### Measurement of Growth Parameters and Transpiration Rate

To assess the growth parameters of tobacco plants, shoots and roots were carefully separated. Fresh mass was measured by weighing the shoots and roots immediately after separation. To determine dry mass, the shoots and roots were dried at 60 °C for 48 h and then weighed.

The leaf transpiration rate was determined by a gravimetric method.^[^ ^33^ ^]^ To minimize water loss from the surface, the plants and hydroponic solution were transferred into a pot covered with aluminum foil. The pot–plant system was weighed and was referred to as *W*_0_. The pot–plant systems were then weighed every 30 min for a total duration of 5 h, with the final weight referred to as *W*_f_. The leaf transpiration rate was calculated as: 600 000 × (*W*_0_ − *W*_f_)/*t* × *A*, where *t* is the time in min, and *A* is the leaf area in cm^2^. Leaf area, root surface area and root total length were calculated as follows: leaves of a whole plant were detached and scanned (Epson perfection V850 Pro, Seiko Epson Corporation, Tokyo, Japan). Leaf area was measured by detaching all leaves from the plant and scanning them using an Epson Perfection V850 Pro scanner (Seiko Epson Corporation, Tokyo, Japan). Root measurements (root surface area and total root length) were obtained using the same scanning method.

### Assessment of the Activity of Antioxidant Enzymes

Protein extractions utilized to analyze CAT, SOD and GSH activities were acquired using the kits provided by Servicebio (China) in line with experimental protocols. APX, PAL, PPO, GR, POD activities were using the activity kit (Nanjing Jiancheng Biotech Inst, China).

### Analysis of Bulk RNA Sequencing

The whole tobacco root of control and CNS treated samples were harvested, and frozen immediately for bulk RNA sequencing by the Illumina Novaseq 6000 sequencing platform. The clean reads were aligned to the reference genome^[^ ^34^ ^]^ using HISAT2 (v2.2.1),^[^ ^35^ ^]^ and the expression levels were calculated and normalized by StringTie (v1.3.3b).^[^ ^36^ ^]^ Finally, differential expression analysis was performed using the DESeq2 package (log2(fold change) > 1, q < 0.05) between different samples.

### Protoplast Isolation from Tobacco Roots and scRNA‐Seq Library Construction

After 16 days of CNS treatment, samples of control and CNS treated roots were collected for protoplast preparation. The protoplast isolation was carried out mainly based on previously established protocol.^[^ ^37^ ^]^ Root segments were dipped into freshly prepared enzyme solution (20 mm MES, pH 5.7, 0.25 m mannitol, 20 mm KCl, 1.5% wt/vol cellulase R10, 0.4% wt/vol Macerozyme R10, 10 mM CaCl2, and 0.1% BSA) for ≈3 h at room temperature. Enzyme‐digested root segments were transferred to MMG solution (4 mm MES, pH 5.7, 0.25 m mannitol and 15 mm MgCl2, room temperature) or modified MMG solution (4 mm MES, pH 5.7, 0.25 M mannitol and 20 mm KCl, room temperature, for subsequent 10x Chromium scRNA‐seq) to release protoplasts. The protoplasts were filtered with 70‐µm nylon mesh, pelleted at 900 g centrifugation for 3 min at room temperature, and resuspended in MMG solution. The isolated protoplasts were examined using a fluorescence microscope, and measurements and statistics were conducted on quality of protoplast. The scRNA‐seq libraries were generated using the 10x Chromium Single Cell 3′ Platform according to the 10X Genomics Chromium Single Cell protocol and sequenced by GENE DENOVO company, followed by quality control of the raw sequencing data.

### scRNA‐seq Data Pre‐Processing

The clean scRNA‐seq reads from control and CNS‐treated samples were separately aligned to the *Nicotiana tabacum* reference genome (NtaSR1),^[^ ^34^ ^]^ using the Cellranger (10x Genomics, v6.1.0) pipeline with default parameters. The gene‐cell matrices were then loaded into the Seurat package (v5)^[^ ^36^ ^]^ for further analysis. To remove the low‐quality genes and cells, only the genes that were expressed in at least three cells were considered, and the cells were filtered with expressed genes more than 5000 or fewer than 200. Cells with more than 1% mitochondrial genes were also filtered out. Two scRNA‐seq datasets from control and CNS treated samples were integrated into an experiment‐wide gene‐cell matrix for subsequent analysis, using harmony method^[^ ^38^ ^]^ in the Seurat package.

### Cell Clustering and Cell Type Annotation

The integrated gene‐cell matrix was normalized by “LogNormalize” in Seurat. To mitigate the effects of cell‐cycle heterogeneity on cell clustering, the cell cycle score for each cell was calculated by the “CellCycleScoring” function implemented in Seurat. The cell cycle‐related genes were identified using homologous genes with Arabidopsis. Then, the highly variable genes were identified using “FindVariableFeatures” with “selection.method = “vst”” parameter, and the top 2500 genes were used for linear dimensionality reduction by principal component analysis (PCA). The first 25 principal components (PCs) were used for a graph‐based clustering, which were further visualized and explored by t‐SNE and UMAP. Cluster‐enriched genes were also identified using “FindConservedMarkers” with parameters “min.pct = 0.25” and “logfc.threshold = 0.58”. Cluster‐specific marker genes were finally selected from these cluster‐enriched genes. Based on the expression patterns of marker genes from PCMDB,^[^ ^39^ ^]^ cell types of each cluster were assigned and determined.

### Interspecies Single‐Cell Data Comparison between Arabidopsis and Tobacco

The scRNA‐seq root data of Arabidopsis was downloaded from NCBI GEO database (accession number GSE167135) as single‐cell transcript abundance matrices.^[^ ^40^ ^]^ Ortholog genes between Arabidopsis and tobacco were identified by OrthoFinder,^[^ ^41^ ^]^ and only one‐to‐one orthologous genes were retained for subsequent analysis. Then, IntegrateData, ScaleData, and RunPCA function were applied to integrate the two datasets for further analysis. Metaneighbor^[^ ^42^ ^]^ was used to measure the correlation between each pair of cross‐species cell clusters.

### Construction of Co‐Expression Networks

To understand the co‐expression relationships between genes at a systems level, high‐dimensional weighted gene co‐expression network analysis (hdWGCNA)^[^ ^43^ ^]^ was utilized to inference gene regulatory network responsive to CNS. hdWGCNA was performed on normalized gene expressed in at least 5% cells. Briefly, irst the data was aggregated from 20 cells in the same cluster to make pseudo‐cells for each cell type, and then constructed a gene co‐expression matrix and groups of closely co‐expressed genes into modules. The hub genes for each module were identified as module eigengene based connectivity kME>0.8 and *P* < 0.05. The randomForest package^[^ ^44^ ^]^ in R was used to assess the accuracy of module classification, and Cytoscape (v3.7.2)^[^ ^45^ ^]^ was used to visualize the co‐expression networks of specific modules.

### Pseudotime Trajectory Analysis

To explore the developmental trajectories of specific cell clusters, the R package Monocle2 (v2.10.0)^[^ ^46^ ^]^ was used. Briefly, the subset for target cell clusters were extracted, and the variance between gene's expression across cells was calculated using “dispersionTable”. The normalized gene expression data by FindVariableGenes function in Seurat were used to define a developmental progress. The dimensionality of the data was subsequently reduced to two components using the “DDRTree” method, and the trajectory was inferred with the “orderCells” function. The pseudo‐time trajectory was then plotted using the “plot_cell_trajectory” function in Monocle2. Finally, “BEAM” was used to identify the pseudo‐time‐dependent genes.

### Evolutionary Analysis of AQPs Family

The phylogenetic trees were constructed based on the protein sequences for each aquaporins. Multiple sequence alignment and neighbor‐joining (NJ) method were used to build phylogenetic trees with a bootstrap value of 1000 in MEGA X (Kumar et al., 2018). The output trees were visualized by ggtree.^[^ ^47^ ^]^

### Vector Construction and Gene Silence

According to the designed reverse fragment primer, cDNA from tobacco (*N. tabacum*, cv. K326) was used as a template to amplify the interference fragment. The interference fragment was inserted into the pCambia2301‐KY‐RNAi vector via BamHI/XbaI digestion and ligated with homologous recombinase. The ligation mixture containing the recombinant vector was introduced into *Escherichia coli* DH5α competent cells. Positive transformants were selected as intermediate vectors. Subsequently, the interference fragment was amplified with forward fragment primers and cloned into the intermediate vector via KpnI and SacI digestion. The reaction product was also transformed into *E. coli* DH5α competent cells, from which plasmid DNA was extracted to obtain the final interference vectors. This procedure was repeated for the construction of vectors for *TIP2;1*, *TIP1;1*, *POT8*, and *NRT3;1*. The primers used in this study are listed in Table S12 (Supporting Information).

## Conflict of Interest

The authors declare no conflict of interest.

## Author Contributions

J.J.J. and P.J.C. conceived and designed the experiments. L.T.C., P.L., H.S., and J.M.T. performed bioinformatics data analysis. L.T.C., J.F.Z., and Z.C.Q. did molecular experiments. L.T.C. and T.B.L. prepared fluorescein‐labeled carbon nanosol material. L.T.C., L.W., and Q.S.C. prepared *TIP2;1* and *TIP1;1* RNAi mutants. L.T.C., L.W., W.Z., N.L., P.J.C., and J.J.J. wrote the manuscript and all authors read and approved the final version.

## Supporting information



Supporting Information

Supplemental Tables

## Data Availability

The raw sequence data reported in this paper have been deposited in the Genome Sequence Archive in BIG Data Center, Beijing Institute of Genomics (BIG), Chinese Academy of Sciences, under accession numbers CRA016403, which can be publicly accessible at http://bigd.big.ac.cn/gsa.
