# Supplementary material for: Single‐Cell Transcriptome Reveals Aquaporin‐Mediated Carbon Nanosol‐Induced Growth Promotion of Plants
Source: Adv Sci (Weinh). 2025 Apr 30;12(27):2504459. doi: 10.1002/advs.202504459 (PMC12279195; doi:10.1002/advs.202504459)
Supplement: Supplementary file 1 — Supporting Information [file ADVS-12-2504459-s002.docx]

**Single-Cell Transcriptome Reveals Aquaporin-Mediated Carbon Nanosol-Induced Growth Promotion in Tobacco**

Lingtong Cheng^1,2^, Zechao Qu^2^, Qiansi Chen^2^, Lin Wang^2^, Huan Su^1,2^, Jiemeng Tao^1,2^, Peng Lu^1,2^, Taibo Liang^3^, Jianfeng Zhang^1,2^, Peijian Cao^1,2*^, Jingjing Jin^1,2*^

^1^Beijing Life Science Academy, Beijing 102200, China

^2^China Tobacco Gene Research Center, Zhengzhou Tobacco Research Institute of CNTC, Zhengzhou 450001, China

3Key Laboratory of Ecological Environment and Tobacco Quality, Zhengzhou Tobacco Research Institute of CNTC, Zhengzhou 450001, China

^*^Correspondence: peijiancao@163.com; jinjingjing1218@126.com

**
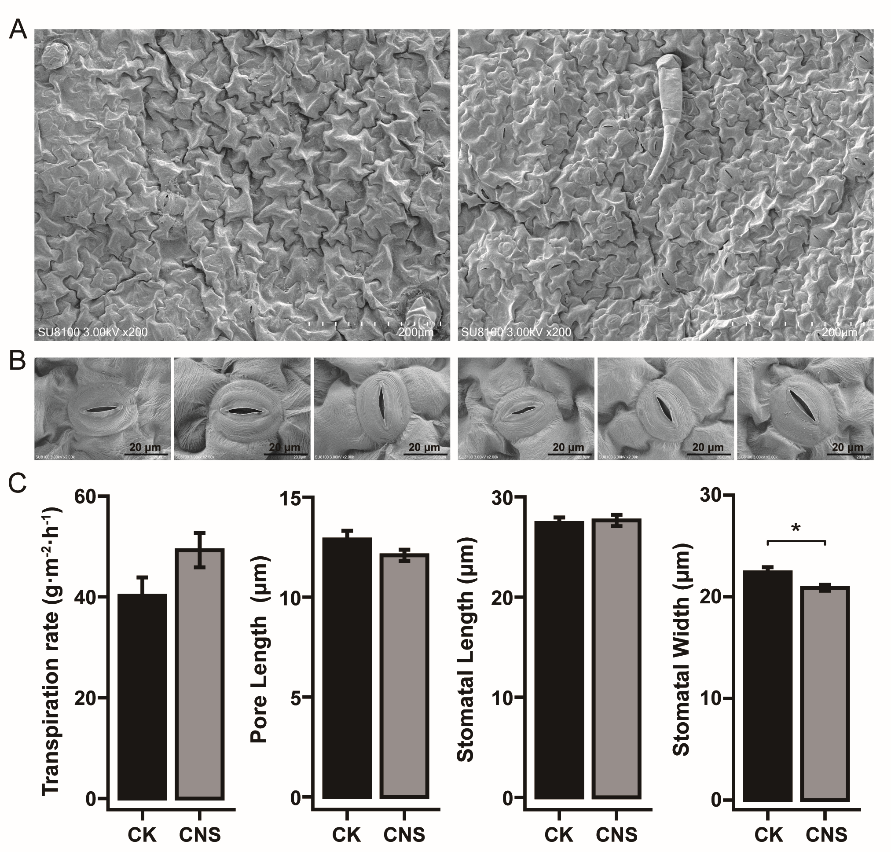
**

**Figure S1. Electron micrographs of stomata cells in adaxial face for CNS-treated tobacco leaves.**

(A) Representative TEM images of *N. tabacum* leaves of control plants and CNS-treated plants after 16 days. Scale bars = 200 µm.

(B) All sections were observed under SEM at 1,500× magnification. Scale bar = 20 μm.

(C) Effects of CNS treatment on transpiration rate and stomatal aperture of adaxial (upper surface of leaf) stomata. Values are the mean ± SD. * - extremely significant difference (*P* < 0.05).

**
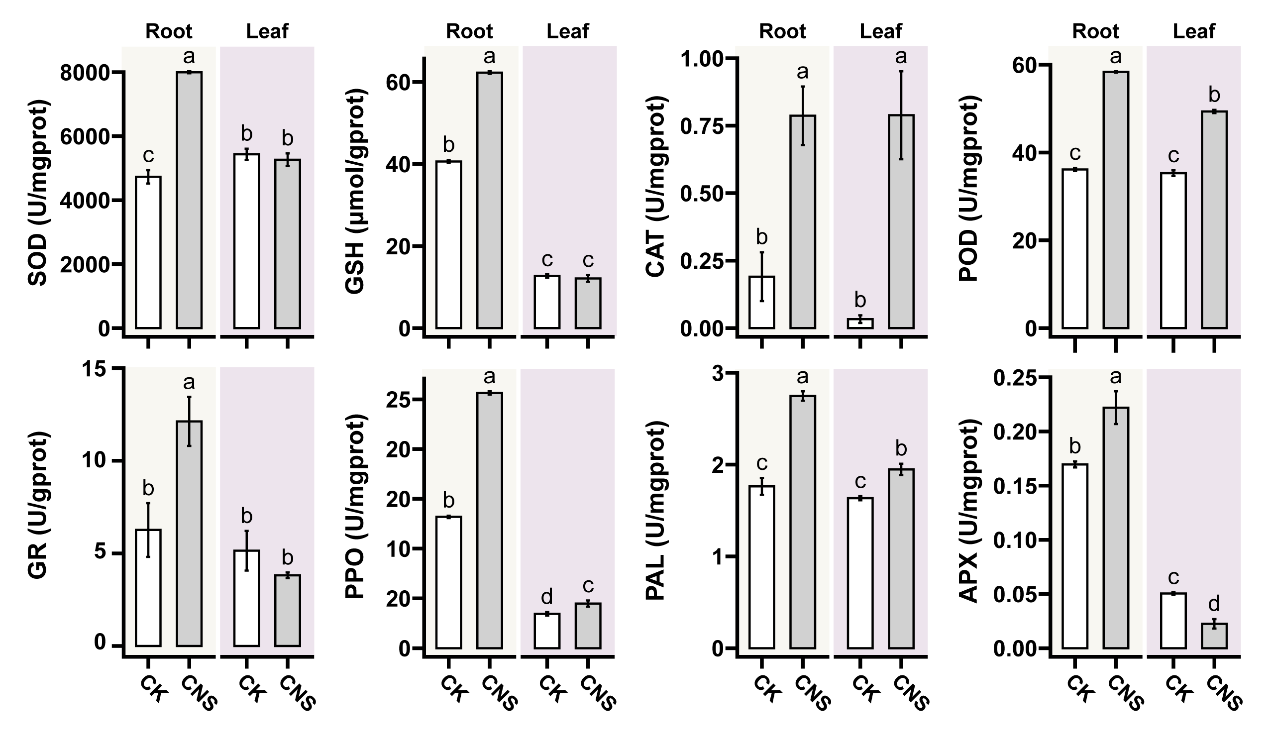
Figure S2.** Enzymatic activity of of tobacco roots and leaves after 16 days of 10 mg/L CNS treatment, including superoxide dismutase (SOD), Glutathione (GSH), Catalase (CAT), Peroxidase (POD), gluathione reductase (GR), Polyphenol oxidase (PPO), phenylalanine ammonia-lyase (PAL), ascorbate peroxidase (APX). Significant differences were assessed using one-way analysis of variance (ANOVA) with Tukey's HSD test. Different letters indicate statistically significant differences between samples (p ≤ 0.05).





**Figure S3. The relative content of** IAA and ABA in tobacco roots after 16 days of 10 mg/L CNS treatment. Statistically significant differences were calculated by the T-test (** indicates P < 0.01, *** indicates P < 0.001), when compared with control (CK).

**
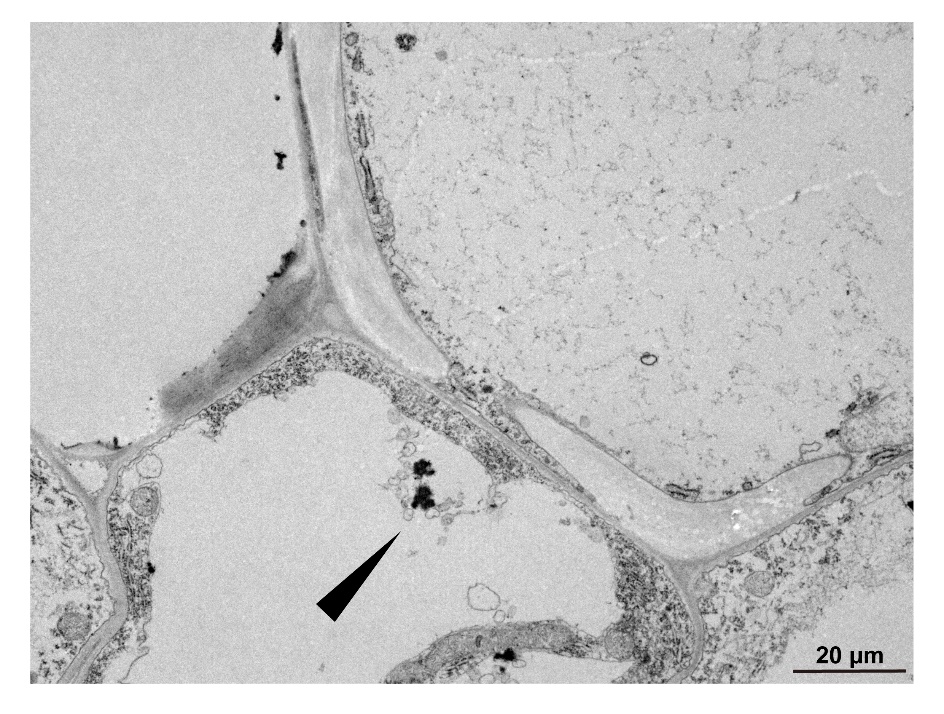
Figure S4. Electron micrographs of CNS-treated *Nicotiana tabacum* root cells.**

Representative TEM images of *N. tabacum* plants 16 days post-treatment with CNS. CNS (Solid arrows) were predominantly found inside the root cells. Scale bars = 20 µm.


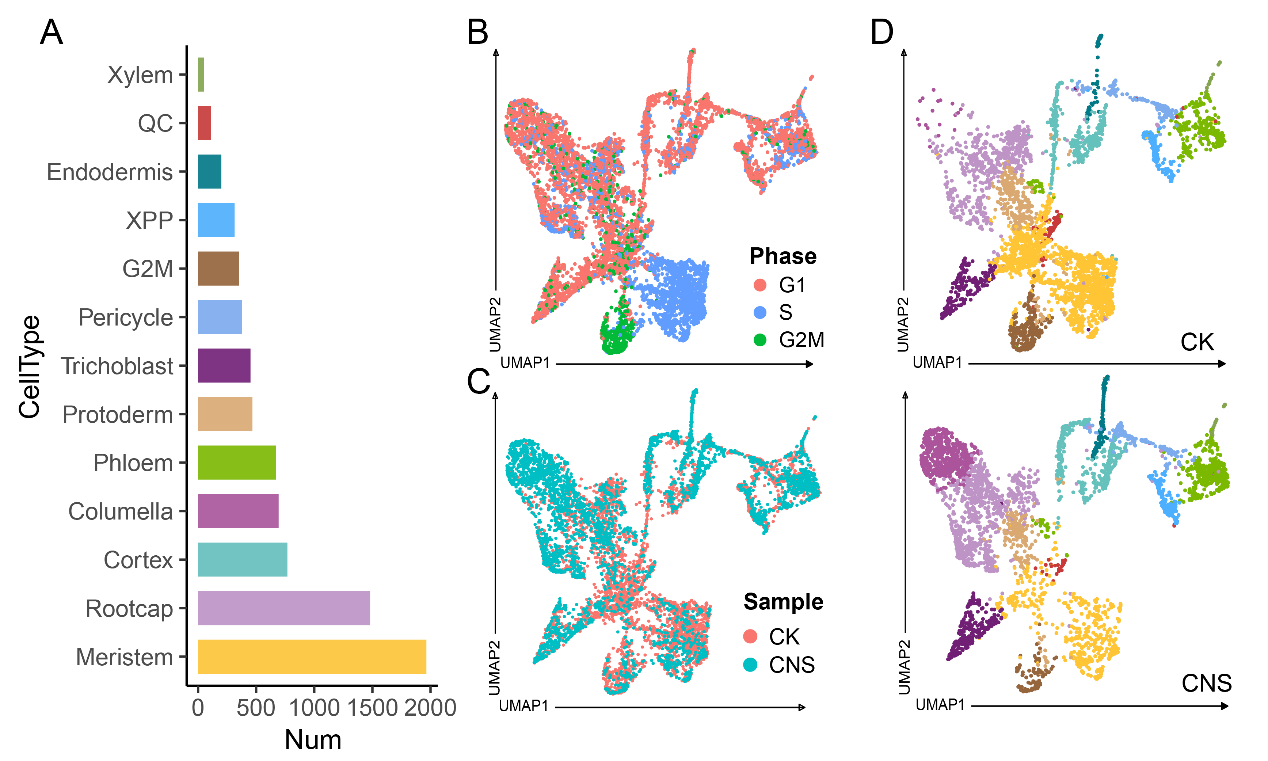


**Figure S5.** Influence of CNS treatment on cell cycle, and cell type of tobacco roots. (A) The number of different cell types in tobacco roots. (B) UMAP dimensionality reduction plot of single cells from tobacco roots colored by cell cycle phases. (C) UMAP dimensionality reduction plot colored by sample origin. (D) UMAP dimensionality reduction plot colored by different cell types.

**
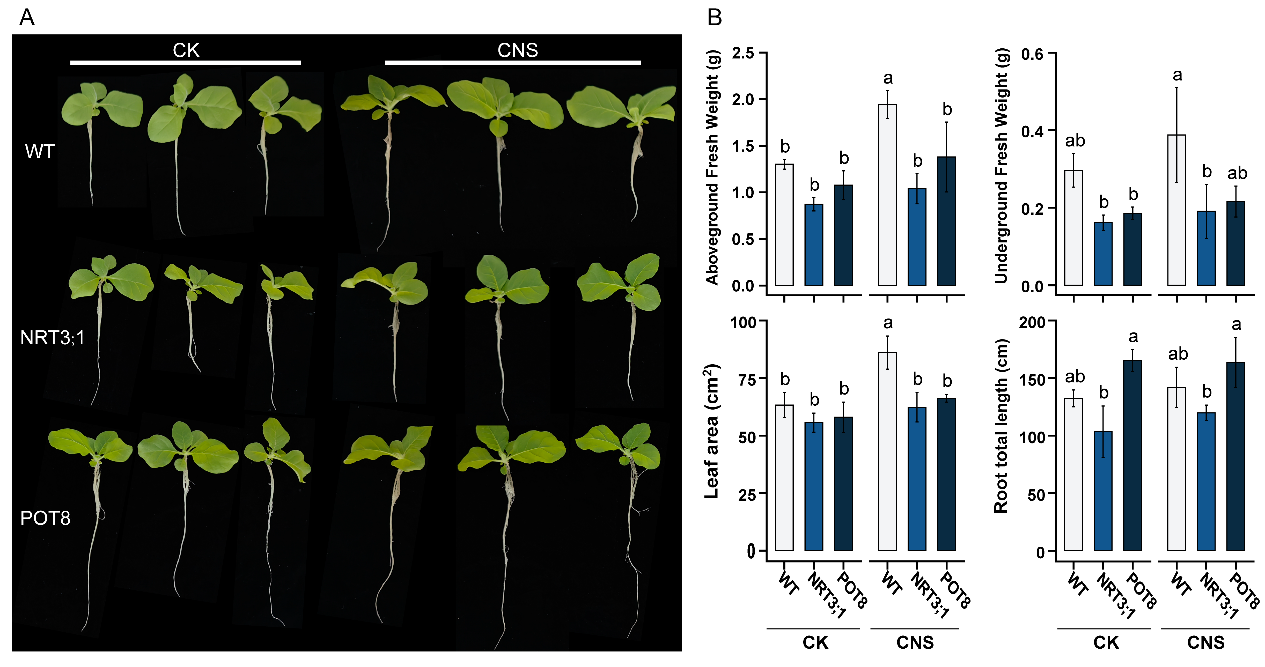
Figure S6.** The growth promotion effect of CNS for tobacco on different nutrient transport (*NRT3.1* and *POT8*) mutants. (A) The phenotype was obtained from plants cultivated under control (CK) and CNS treatment. Seedlings of wild type (Col-0), *NRT3.1* RNAi line (*nrt3.1*) and *POT8* RNAi line (*pot8*) were grown in nutrient solution containing 10 mg/L CNS. (B) The length of roots, leaf size and biomass were obtained when the tobacco plants were treated for 10 days. Error bars depict the SD from 5 randomly selected plants. Different letters on the columns indicate a significant difference of *P* < 0.05 by Duncan test.

**
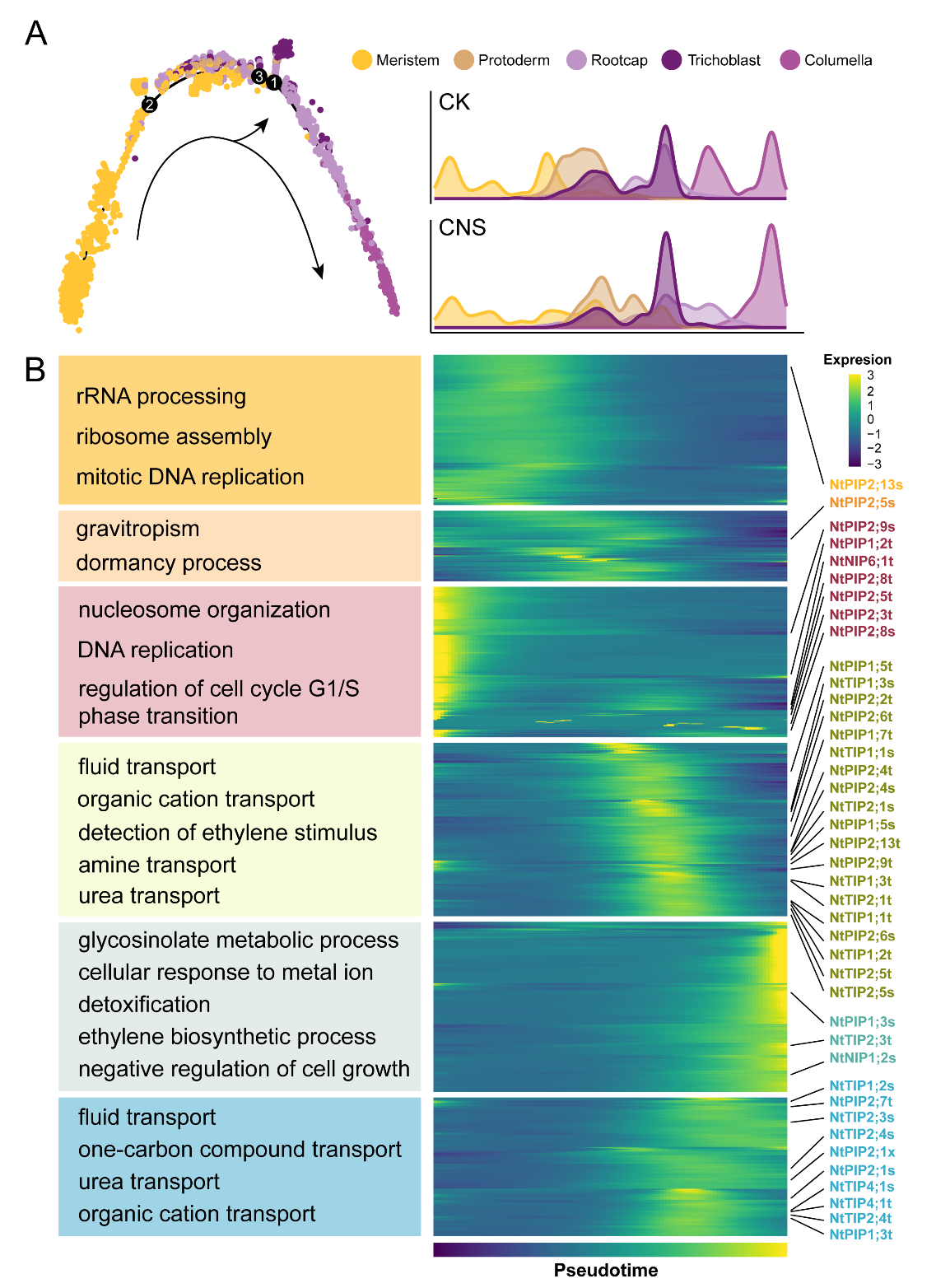
Figure S7. Differentiation trajectory of control and CNS-treated epidermal cells.**

**(A)** Differentiated trajectories of trichoblast, root cap and columella. **(B)** Heatmap showing the expression of differentially expressed genes for the epidermal cells over pseudotime. Color bar indicates the relative expression level.
